# A Correlation Between Differentiation Phenotypes of Infused T Cells and Anti-Cancer Immunotherapy

**DOI:** 10.3389/fimmu.2021.745109

**Published:** 2021-09-15

**Authors:** Hao Ren, Kunkun Cao, Mingjun Wang

**Affiliations:** Department of Research and Development, Shenzhen Institute for Innovation and Translational Medicine, Shenzhen, China

**Keywords:** memory T cells, phenotypic molecules, differentiation, clinical trials, anti-cancer immunotherapy

## Abstract

T-cell therapy, usually with ex-vivo expansion, is very promising to treat cancer. Differentiation status of infused T cells is a crucial parameter for their persistence and antitumor immunity. Key phenotypic molecules are effective and efficient to analyze differentiation status. Differentiation status is crucial for T cell exhaustion, in-vivo lifespan, antitumor immunity, and even antitumor pharmacological interventions. Strategies including cytokines, Akt, Wnt and Notch signaling, epigenetics, and metabolites have been developed to produce less differentiated T cells. Clinical trials have shown better clinical outcomes from infusion of T cells with less differentiated phenotypes. CD27+, CCR7+ and CD62L+ have been the most clinically relevant phenotypic molecules, while Tscm and Tcm the most clinically relevant subtypes. Currently, CD27+, CD62L+ and CCR7+ are recommended in the differentiation phenotype to evaluate strategies of enhancing stemness. Future studies may discover highly clinically relevant differentiation phenotypes for specific T-cell production methods or specific subtypes of cancer patients, with the advantages of precision medicine.

## Introduction

T-cell therapy is a very promising anti-cancer therapy. It includes ex-vivo expansion and reinfusion of tumor-reactive T cells such as tumor-infiltrating lymphocytes (TILs) and, genetically-engineered T cells with conventional T-cell receptors (TCRs) or chimeric antigen receptors (CARs). CD19-specific CAR-T cell immunotherapy has achieved a complete response rate of more than 90% in B-cell leukemia and 30-50% in lymphoma (1). The efficacy of NY-ESO-1-specific TCR-T cell therapy in synovial cell sarcoma and melanoma has been up to 61% and 55%, respectively (1, 2). However, CAR-T cell therapy is not very effective in treating solid tumors. NY-ESO-1-specific TCR-T therapy is useful in just a few solid tumors. And even in these just a few solid tumors, the efficacy is still not satisfactory (1, 2). Therefore, tumor immunotherapy is still facing big challenges in the treatment of solid tumors (2).

Differentiation status of infused T cells is emerging as a crucial parameter for generating cell products with superior persistence and antitumor immunity (3, 4). The phenotype is a both effective and efficient indicator to analyze differentiation status of T cells. Quite a few clinical trials have shown that infused T-cell differentiation phenotypes are significantly relevant to clinical outcomes in anti-cancer therapies (5–11). However, the specific differentiation phenotypes indicating the strongest T-cell therapeutic effects are still controversial. Here, we summarize the clinical investigations on differentiation phenotypes of infused T cells and their anti-cancer therapy, which may provide some insights to production of T cells with good quality for cancer immunotherapy.

## Phenotypic Molecules of T Cell Differentiation

The clinical trials revealed several T-cell differentiation phenotypes for anti-cancer therapies (5–11). The key molecules used to identify the phenotypes include CCR7, CD27, CD28, CD39, CD45RA, CD45RO, CD62L, CD69, CD95, IL7Rα.

### CCR7

CC Chemokine Receptor 7 (CCR7), also known as CD197, is a kind of CCR receptors with specificity for chemokine CCL19 and chemokine CCL21. CCR7 regulates the homing of T cells to lymphoid organs. Controlling the migration of memory T cells by CCR7 modulation may determine their destiny by changing the location (12). In addition to controlling cell migration, CCR7‐mediated signals affect T‐cell homeostasis in lymph nodes and also influence T‐cell activation and polarization (13).

Depending on the subtypes or functions of memory T cells, CCR7 may also determine the niche suitable for their survival. So CCR7 plays a crucial role in the activation of naïve T cells (Tn) as well as the development and maintenance of memory T cell subsets (12).

### CD27

CD27, also known as T-Cell Activation Antigen or TNFRSF7 Receptor (Tumor Necrosis Factor Receptor Superfamily, Member 7), is a member of the tumor necrosis factor receptor superfamily found on T-lymphocytes. It is a T cell co-stimulatory immune-checkpoint receptor (14), activated by the transient availability of its TNF-like ligand CD70 on lymphocytes and dendritic cells. This activation results in the increased proliferation of T cells. Key contributions of the CD27-CD70 system have been revealed on effector and memory T cell formation, which is probably based on improved cell survival (15). Co-stimulation of CD8^+^ T-cells through CD27 also enhances primary, secondary, memory and recall responses (14). An agonistic antibody has entered clinical trials for cancer immunotherapy (16).

### CD28

CD28, also known as TP44 Receptor, is a kind of costimulatory T-lymphocyte receptors with specificity for CD80 antigen and CD86 antigen. Activation of T lymphocytes requires at least two signals, one delivered by TCR complex after antigen recognition, and one by co-stimulatory receptors, such as CD28 (17). CD28 also acts as a TCR-independent signaling unit to regulate the expression of pro-inflammatory cytokines (18). CD28 activation promotes T-cell proliferation, cytokine production, T-cell survival. Its stimulation also prevents clonal inactivation or anergy (19).

CD28 loss on T cells is the most consistent biological indicator of immune incompetence in aging. There is also mounting evidence for the high frequency of CD28^-^ T cells among patients with inflammatory syndromes or with chronic infections disproportionate with their age (20), as well as in multiple solid and hematogenous tumors (21). They are derived from CD28^+^ precursors that have undergone repeated stimulation, indicating that CD28 silencing underlies the program of T-cell aging (20). CD28^-^ T cells are very heterogeneous. Among them certain populations seem to promote allograft tolerance whereas others contribute to alloreactivity and costimulation blockade resistant rejection (22).

### CD39

CD39, also known as ecto-apyrase, ectonucleoside triphosphate diphosphohydrolase 1, NTPDase-1, ectoADPase or ecto-ATP diphosphohydrolase, belongs to the GDA1/CD39 NTPase family. It is expressed primarily on activated lymphoid cells, and also in endothelial tissues. It hydrolyzes extracellular adenosine triphosphate (ATP), adenosine diphosphate (ADP) and adenosine monophosphate (AMP) into adenosine. CD39 modulates immunity (23). Its expression is induced on T cells and increased on B cells as a late activation antigen. It is a marker for terminally exhausted CD8+ T cells in patients with chronic hepatitis C virus and human immunodeficiency virus infections (24), and for exhaustion in tumor-infiltrating CD8+ T cells in melanoma and breast cancer (25). CD39+ neoantigen-specific TIL subsets are likely terminally differentiated TILs with poor proliferation, likely due to chronic antigenic stimulation.

As a cell surface marker, CD39 showed low expression in T-cell clusters associated with response in immunotherapy, and high expression in those associated with no response (26). Tumor control was enhanced when targeting CD39 in combination with anti-TIM3, PD-1 or PD-1/CTLA4 (27).

### CD45

CD45, also known as T200, B220, 2H4 or leukocyte common antigen, is a kind of glycoproteins that constitute much of the cell surface of lymphocytes (28). Its cytoplasmic domain possesses protein tyrosine phosphatase activity (29). Its multiple isoforms result from alternative mRNA splicing of exons 4, 5 and 6, generating extracellular domains A, B, and C respectively (30). There are at least eight possible isoforms of CD45, but only RO (containing no alternatively spliced domains), RA, RB, RBC, and RABC polypeptides are found on human and murine lymphocytes (31).

Tn express the high molecular weight isoform CD45RA and that is lost after activation and replaced by the low molecular weight isoform CD45RO (32). Then T cells may also revert from CD45RO^+^ to CD45RA^+^ (33). Persistent viral infections, inflammatory syndromes and aging all induce the accumulation of highly differentiated CD45RA re-expressing memory T cells with decreased proliferative capacity, increased activation of senescence signaling pathways, greater susceptibility to apoptosis, as well as multiple effector functions (34).

### CD62L

CD62L, also known as Mel-14, Leu-8, TQ1, L-Selectin, or Leukocyte Adhesion Molecule-1, is a homing receptor for Tn, stem central memory T cells (Tscm) and central memory T cells (Tcm) and to lymph node high endothelial venules (35). Tscm and Tcm, which lack immediate effector function but rapidly proliferate in secondary responses, express CD62L and are found in lymph nodes, whereas effector memory T cells with immediate effector function, lack CD62L and are found in blood, peripheral tissues and spleen but not in lymph nodes (36, 37).

### CD69

CD69, also known as activation inducer molecule, early activation antigen EA1, Leu-23, C-type lectin domain family 2 member C, or p60, is a single-pass type II membrane protein. CD69 is one of the earliest markers up-regulated after T cell activation (38), and has been regarded as an activation marker (39). It is involved in lymphocyte proliferation and functions as a signal transmitting receptor in lymphocytes, natural killer cells, and platelets. CD69 is also critical for the generation and maintenance of professional memory Th lymphocytes, which can efficiently help humoral immunity in the late phase (40). CD69 functions as a homing receptor on CD4 T cells through S1P_1_, which regulates cell trafficking (41). CD69-overexpressing thymocytes accumulate in the thymus and do not enter the periphery (42, 43).

In contrast to CD69^-^ terminal effector T (T_TE_) cells, oligoclonal expansions are not evident within CD69^+^ T_TE_ cells, which possess low perforin and granzyme expression and high inhibitory checkpoint expression and resemble T resident memory cells (44). The balance between CD69^-^ and CD69^+^ cells within the bone marrow-T_TE_ compartment may regulate immune responses in new diagnosed multiple myeloma and contribute to the clinical disease heterogeneity.

CD8+CD69+ T-cell prevalence may be an activity biomarker in Crohn’s disease (45). It is more abundant in active patients than in those in remission.

### CD95

CD95, also known as APO-1, FAS, Fas Cell Surface Death Receptor, or TNFRSF6 (Tumor Necrosis Factor Receptor Superfamily, Member 6) Receptor, is a canonical death receptor found in a variety of tissues and on activated lymphocytes. It is engaged in the extrinsic pathway of cell death, leading to the formation of the death-inducing signaling complex (46). In T cells, CD95 plays a major role in activation-induced cell death (47). CD95 also facilitates T-cell differentiation (48). It is important in the control of T cell homeostasis and the contraction of immune responses (46).

### IL7Rα

IL7Rα (Interleukin 7 Receptor alpha Subunit), also known as CD127, is a low affinity IL-7 receptor subunit. The high affinity receptor IL-7R is comprised of this unique α chain and CD132. IL-7 plays a key role in the development of both thymic and peripheral T cells (49). IL7Rα signals support the survival of periphery mature T cells, not only for typical αβ T cells, but also for γδ T cells, and even with a partial role for survival of regulatory T cells (Treg) (50). They maintain homeostasis of naive and memory T cells (51). They maintain Tn in an interphase whereas they induce occasional cell division of memory T cells (52). The interplay between TCR and IL-7R regulates IL-7R expression (50).

## T Cell Differentiation Status and Antitumor Responses

Differentiation status is crucially related to T cell exhaustion, in-vivo lifespan, and antitumor immunity. Differentiation status even impacts effects of antitumor pharmacological interventions.

### T Cell Exhaustion

The transferred tumor-specific memory cells have become exhausted TILs with extensive heterogeneity in tamoxifen-induced autochthonous hepatocellular carcinoma (53) and fibrosarcoma (MCA205) (54) mice. Different cellular subsets show distinct cellular markers and functional capabilities including self-renewal, proliferation and immunoresponse (**Table 1**). It implies differentiation status, which is the significant difference between these subsets, can play an important role in T cell lifespan and immunotherapy.

**Table 1:** Extensive heterogeneity in exhausted T cell populations.

Exhausted T cells	Cellular markers							Self-renewal and proliferation	Response to checkpoint inhibition
	PD1	T-bet	TIM3	CD38	CD101	CXCR5	others		
Memory-like	mid	hi	low	low	low	+	TCF1^hi^	High potential	Responsive
Differentiated	hi	low	hi	hi	hi	–	EOMES^hi^	Low potential	Resistant

### Differentiation Status Regulates T Cell Lifespan

After initial antigen encounter, naïve T cells are activated, rapidly proliferate and then produce cytokines and granules (55), which is a course of differentiation with progressive change in transcription factor expressions (56), epigenetics and metabolism (57). The less differentiated Tn, Tscm and Tcm cells have increased longevity and replicative capacity compared with effector memory T cells (Tem), effector T cells (Teff) and terminally differentiated Temra (58). Also, increased T cell differentiation has repressed telomere length (59) and transcription factors promoting self-renewal and cell longevity (56).

### Less Differentiated T Cells Against Tumors

T-cell persistence is one of the key determinants for immunotherapy efficacy (58). Minimally differentiated T cells with enhanced self-renewal ability have mounted superior immune responses against established B16 melanoma in mice (60). Human-derived mesothelin-specific Tscm have shown more enhanced antitumor activity than Tcm and Tem in M108 mesothelioma mice (61). In melanoma patients, TILs exhibiting effective antitumor responses also have better persistence and survival *in vivo (* 62). Less differentiated Tn, Tscm and Tcm cells have been more effective against established vascularized B16 melanoma in mice than Tem or Teff (63). Respectively, Tscm cells have shown more efficacious reconstitution in immunodeficient hosts (61). Tcm cells have stronger cytotoxicity (64), persisted longer in macaques (65) and humans (Clinicaltrials.gov ID: NCT03575806). Memory cell markers have also clinically been found closely related with telomere length and antitumor responses in metastatic melanoma patients (9, 66). Besides persistence, the superior antitumor efficacy of less differentiated T cells may also be because of their enhanced capacity to migrate to lymph nodes with homing receptor CCR7, primary immunosurveillance of peripheral tissues (3), and relatively weak inhibition by Tregs (67).

### Differentiation Status Impacts Effectiveness of Pharmacological Interventions

Pharmacological intervention effects can also depend on the differentiation status of T cells. S-2-hydroxyglutarate (S-2HG) improves memory expansion and T-cell therapy in EG7-OVA tumor mice only when administered to naive T cells, and not when administered to the T cells with a more differentiated state (68). Not only the effect of pharmacological interventions, desirable therapeutic effects in one subset may also be prohibitive in another of different differentiation status. To sum up, low differentiation status of T cells is crucial for antitumor therapy.

## Strategies to Produce Less Differentiated T Cells

Given the importance of the less differentiated T cells for antitumor therapy, strategies have been developed to produce them.

### Cytokines

Cytokines IL-7 and IL-15 have been shown to promote the generation of Tcm-like cells (69). Each of them alone has also increased memory phenotypes (3). What is more, IL-7-expressing CAR-T cells have achieved complete regression of solid tumors (70). IL-7 receptor signaling has resulted in prosurvival in memory cells, while IL-15 positively affected mitochondrial (71) and catabolic metabolism (58).

Th9-culture condition using IL4 and TGF-β has enhanced proliferation, central memory phenotype and antitumor activity (72). 3-day IL-4 exposure during TCR stimulation has also induced long-lasting memory T cells (73). The mixture of IL-4, IL-7 and IL-21 has promoted central memory and stem cell memory phenotypes, and lowered inhibitory receptors PD-1, LAG-3 and TIM-3 in CAR19 T cells (74).

### Akt, Wnt, and Notch Signaling

Controlling Akt/mTOR signaling has also driven a memory phenotype (75). Rapamycin has exerted an immunostimulatory effect to generate memory CD8+ T cells (76) including Tcm and Tem, and even impacted proliferation (75). Tacrolimus has also promoted Tcm cells. Akt inhibition has reduced T cell glycolysis, enhanced the expansion of both CD4+CD62L+ and CD8+CD62L+ memory TILs, promoted mitochondrial metabolism and antitumor properties (77). Fas signaling has also been implicated in Akt-driven T-cell differentiation (78). Impairment of TCR-driven Akt-mTOR phosphorylation by potassium has disrupted effector function and tumor clearance (79).

Glycogen-synthase-kinase-3β inhibitor TWS119 has modulated Wnt signaling, blocked T cell differentiation, enhanced metabolic fitness (80), promoted self-renewing memory stem cells with proliferative and antitumor capacities (60).

Tscm can be generated from not only Tn, but also activated T cells. Coculturing with stromal cells expressing a Notch ligand has generated Tscm-like cells *in vitro* from activated mouse and human T cells (81).

### Epigenetic Strategies

Conditional knockout of Dnmt3a in T cells has promoted the less-differentiated subsets with increased T-bet and TCF1 as well as decreased TIM3 and EOMES, thus enhanced persistence and cytokine production (82). Epigenetic strategies also include induced pluripotent stem cells (iPSCs). Transient expression of octamer-binding protein 4, SOX2, Krüppel-like factor 4 and MYC has reprogrammed T cells into iPSCs through stage-specific transcription factors, histone-modifying proteins, and chromatin-remodeling enzymes (83–87). Differentiated T cells may also be directly reprogrammed into less-differentiated ones (88), aiming at the silenced state of T cell-specific stemness and memory genes (89).

Bromodomain inhibitor JQ1 has inhibited effector differentiation by epigenetic modifying proteins, inhibited the histone acetylation reader BRD4 and histone deacetylase SIRT1, enhanced memory formation and persistence, and finally promoted antitumor activity (90). Metabolic by-product S-2HG has also targeted epigenetic proteins, inhibited effector differentiation, and promoted therapy (68). It has inhibited α-ketoglutarate-dependent proteins, such as the Jumonji family of histone demethylases and the TET family of DNA hydroxylases (91). C646 has inhibited the histone acetyltransferase p300, increased both CD8+CD45RA+CD62L+CCR7+ and CD8+CD45RA-CD62L+CCR7+ T cells, and eventually augmented antitumor effects (90).

Transcription factor c-Myb overexpression has enhanced CD8+ T cell memory formation, polyfunctionality and recall responses that promoted curative antitumor immunity in B16 tumor mice (92).

miR-155 has increased CD8+ T cell antitumor function by restraining T cell senescence and functional exhaustion through epigenetic silencing of drivers of terminal differentiation (93). miR-155 has promoted antitumor responses in melanoma mice through miR-155-Phf19-Polycomb repressor complex 2 axis.

### Metabolites

Direct inhibition of hexokinase by 2-deoxyglucose has promoted CD62L+ memory cells and antitumor efficacy against established and vascularized melanoma (94). L-arginine has modulated T cell metabolism, promoted CD4+CCR7+ cells, antitumor activity and survival (95). Lactate dehydrogenase inhibition combined with IL-21 has promoted CD8+ T cell stemness and antitumor immunity in melanoma mice (96).

### Others

Mitochondrial division inhibitor 1 has promoted fused mitochondrial structures and inhibited mitochondrial fission, increased memory cells, and improved antitumor activity (97).

RORγ agonists have also promoted durable memory and stemness of cells including Th17 and Tc17 (98). Central and tissue-resident memory populations have been conferred by P2RX7 (99), a purinergic receptor sensing extracellular ATP.

## Infusion of T Cells With Less Differentiated Phenotypes Has Better Clinical Outcomes in Antitumor Therapy

**Figure 1:**
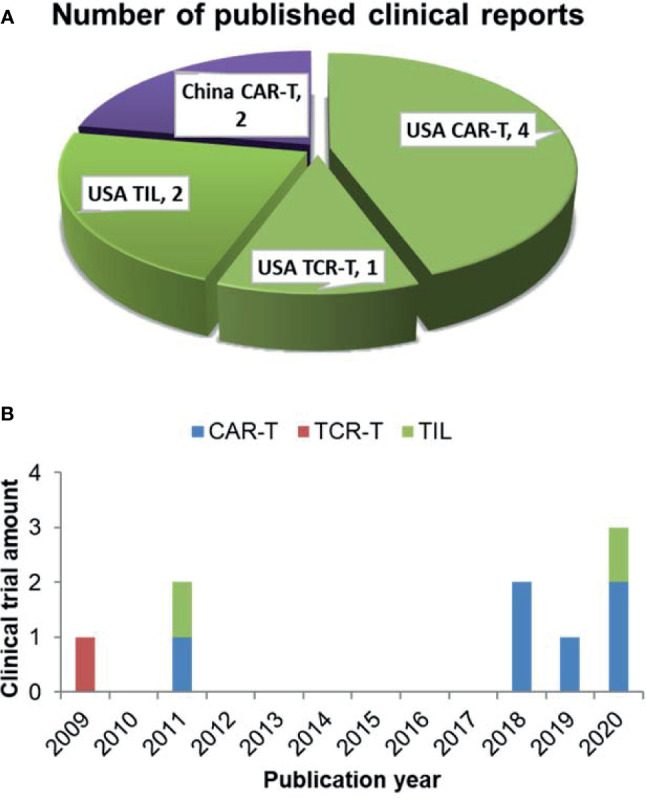
Literature review of clinical trials analyzing differentiation phenotypes of the infused T cells and their anti-cancer outcomes. **(A)** Number of the published clinical reports from different countries (purple for China, green for USA). **(B)** The amount of clinical trials with various T-cell therapy types published in different years.

9 clinical trials have analyzed the correlation between differentiation phenotypes of the infused T cells and their anti-cancer outcomes, with 2 in China and 7 in USA (**Figure 1A**). The first trial has been published in 2009, after which more and more clinical investigators have begun to study this correlation (**Figure 1B**). The characteristics of the trials have been summarized in **Table 2**. The associations of differentiation phenotypes with objective response have also been summarized (**Table 3**).

**Table 2:** Clinical trials on differentiation phenotypes of infused T cells and anti-cancer therapy.

Publication year	Cancer	No. of Patients	T-cell therapy type	Antigen	Construct design	Transduction methods	Manufacture	Transfer dose	Objective response	Registration No.	Reference
2009	Melanoma	36	TCR-T	DMF5	DMF5 TCRα-T2A-DMF5 TCRβ	Retroviral vector	Stimulated with OKT3 (50 ng/mL) and IL2 (300 IU/mL) for 2 days, transduced and expanded with IL2 (6000 IU/mL) for 7-10 days, stimulated and expanded with OKT3, irradiated PBL and IL2 (6000 IU/mL) for 9-14 days	1.5-10^7^×10^9^	1 CR, 8 PR, 27 NR	NCI-07-C-0174, NCI-07-C-0175	(6)
2011	Neuroblastoma	19	CAR-CTL	GD2	GD2-specific 14g2a.ζ CAR	Retroviral vector	Stimulated with OKT3 and IL2 (100 U/mL) for 2 days, transduced, expanded with IL2 (50 U/mL) for 9-15 days	2×10^7^, 5×10^7^ or 1×10^8^ cells/m^2^	3 CR, 8 NED, 1 PR, 1 SD, 4 PD, 2 tumor necrosis	NCT00085930	(7)
2011	Melanoma	93	TIL	N/A	N/A	N/A	Grown from resected metastatic melanoma lesions with IL-2 (6000 IU/mL)	Less than 3×10^10^ to more than 9×10^10^	20 CR, 32 PR, 41 NR	None	(9)
2018	Biliary tract cancer	17	CAR-T	EGFR	EGFR-specific CD137-CD3ζ-CAR	Lentiviral vector	Stimulated with OKT3 (50 ng/mL) and IL2 (500 U/mL) for 2 days, transduced, expanded for 10 days	0.8-4.1×10^6^/kg, 1-3 cycles within 6 months	1 CR, 10 SD, 6 PD	NCT01869166	(10)
2018	Chronic lymphocytic leukemia	38	CAR-T	CD19	CD19-specific CAR with 4–1BB/CD3ζ domains	Lentiviral vector	Stimulated by anti-CD3/28 beads, transduced, expanded for 9-11 days	0.2-7×10^6^/kg interquartile	8 CR, 5 PR (3 PR_TD_), 25 NR	NCT01029366, NCT01747486, NCT02640209	(5)
2019	Multiple myeloma	25	CAR-T	BCMA	anti-BCMA scFv-4-1BB-CD3ζ CAR	Lentiviral vector	Stimulated by anti-CD3/28 beads, transduced, expanded with IL2 for 10-12 days	1-5×10^7^ or 1-5×10^8^	1 stringent CR, 1 CR, 5 very good PR, 5 PR, 5 minimal response, 6 SD, 2 PD	NCT02546167	(8)
2020	Pancreatic carcinoma	14	CAR-T	EGFR	anti-EGFR scFv-CD137-CD3ζ CAR	Lentiviral vector	Stimulated with OKT3 (50 ng/mL) and IL2 (500 U/mL) for 2 days, transduced, expanded for 10 days	1.31-8.9×10^6^/kg, 1-3 cycles within 6 months	4 PR, 8 SD, 2 PD	NCT01869166	(11)
2020	Melanoma	38	TIL	N/A	N/A	N/A	Initially cultured from enzymatic tumor digests and tumor fragments, generated by rapid expansion with irradiated PBMC, anti-CD3 antibody (30 ng/mL), and IL-2 (3000 IU/mL) for 14 days	a single infusion of unselected 1.1×10^8^-1×10^11^	17 CR, 21 NR	NCT00001832, NCT00513604, NCT01319565, NCT01468818, NCT01585415, NCT01993719	(100)
2020	LBCL	24	CAR-T	CD19	anti-CD19 scFv-CD28-CD3ζ CAR	Retroviral vector	Stimulated with anti-CD3 antibody and IL-2, transduced, and expanded	2 × 10^6^ CAR-positive cells/kg	9 CR, 1PR, 13 PD, 1 NE	None	(101)

**Table 3:** Association of differentiation phenotypes with response in clinical trials.

Cancer	Differentiation phenotype	Corresponding subtype	Association with response	Reference
Melanoma	CD27+	Tscm and Tcm	Unsubstantial	(6)
Melanoma	CD28+	All	Unsubstantial	(6)
Melanoma	CD45RA+	Tscm, Teff and Temra	Unsubstantial	(6)
Melanoma	CD45RO+	Tcm and Tem	Unsubstantial	(6)
Neuroblastoma	CD45RO+CD62L+	Tcm	Phenotype percentage - CAR-T persistence (p ≤ 0.055), CAR-T persistence – TTP (p=0.02)	(7)
Melanoma	CD8+CD27+	Tn, Tscm and Tcm	CR *vs*. (PR+NR) p=0.001, (CR+PR) *vs*. NR p<0.001	(9)
Biliary tract cancer	CD45RO+CD62L+CCR7+	Tcm	CR/SD *vs*. PD, p=0.0464	(10)
Chronic lymphocytic leukemia	CD8+CD27+CD45RO-	Tscm	CR/PR_TD_ *vs*. PR/NR p=0.0009	(5)
Chronic lymphocytic leukemia	CD8+CD45RO-CCR7+CD45RA+CD62L+CD27+CD28+IL7Rα+CD95+	CD8^+^ Tscm	CR *vs*. NR p=0.0008, CR/PR_TD_ *vs*. PR/NR p=0.0055, CR *vs*. PR p=0.0121	(5)
Multiple myeloma	CD8+CD45RO-CD27+	Tscm	≥PR *vs*. <PR, p=0.0121	(8)
Pancreatic carcinoma	CD45RO+CD62L+CCR7+	Tcm	PR/SD *vs*. PD p=0.0468	(11)
Melanoma	CD8+CD39-CD69-	Stem-like CD8 T cells	CR *vs*. NR, p<0.01	(100)
Melanoma	CD39-CD69-	Stem-like T cells	CR *vs*. NR, p=0.0096	(100)
LBCL	CD8+CCR7+CD27+	CD8^+^ Tscm and Tcm	CR *vs*. PR/PD, p<2.2×10^-16^	(101)

19 patients with neuroblastoma has been treated with CAR-T cells on 3 dose levels: 2×10^7^ cells/m^2^, 5×10^7^ cells/m^2^ or 1×10^8^ cells/m^2^. To produce CAR-T cells, peripheral blood mononuclear cells (PBMCs) have been activated with anti-CD3 mAb (OKT3) and recombinant human (rh)IL-2, transduced on day 3, expanded with 50 U/ml rhIL-2 added every 3 days, and frozen on day 12-18. 3 patients have shown complete response (CR), and 8 no evidence of disease (NED), 1 partial response (PR), 1 stable disease (SD), 4 progressive disease (PD), 2 tumor necrosis. The percentage of CD45RO+CD62L+ T cells in the infused product has been highly concordant with prolonged detection of transferred T cells within the peripheral blood (7). Each 1-unit increase in the percentage of this phenotype has been associated with a 6.1 increase in the log (duration) of CAR-expressing Epstein Barr-virus specific T cells (CAR-ATCs, p=0.055) and a 6.63 increase in the log (duration) of GD2-CAR expressing activated T cells (CAR-CTLs, p<0.0001) in the T-cell product. And this prolonged detection has been associated with prolongation of Time To Progression, which is the primary parameter for clinical outcomes. The persistence of even low levels of either CAR-ATCs or CAR-CTLs at or beyond 6 weeks has been associated with a significantly longer TTP (p=0.02) in patients with active disease. CD45RO+CD62L+ T cells mainly include the subtype of Tcm.

93 patients with measurable metastatic melanoma have been treated with the adoptive transfer of autologous TILs in conjunction with 720,000 IU/kg IL-2 following a lymphodepleting preparative regimen. TIL doses vary from less than 3×10^10^ to more than 9×10^10^. TILs have been grown from resected metastatic melanoma lesions in high-dose IL-2. 20 patients have achieved CR, and 32 PR, 41 NR (no response). The number of CD8+CD27+ cells infused has been significantly associated with objective response [CR *vs*. (PR+NR) p=0.001, (CR+PR) *vs*. NR p<0.001] (9). The CD8+CD27+ cell number in CR patients is (2.0 ± 0.3)×10^10^, and (1.5 ± 0.2)×10^10^ in PR, (0.8 ± 0.1)×10^10^ in NR. CD8+CD27+ cells include the subtype of Tn, Tscm and Tcm.

19 patients with Epidermal growth factor receptor (EGFR)-positive advanced unresectable, relapsed/metastatic biliary tract cancers have received 1-3 cycles of CAR-T-EGFR cell infusion. The dose range is 0.8-4.1×10^6^/kg. To produce CAR-T cells, PBMCs have been stimulated by 50 ng/mL OKT3 and cultured in GT-T551 medium with 0.5% autologous serum and 500 U/mL rhIL2, transduced by lentiviral particles after 2 days, and further expanded for 10 d in culture bags. 2 patients have lost follow-up. Of the 17 evaluable patients, 1 achieved CR for 22 months and 10 had SD for 2.5-15 months from the first cycle of treatment, the other 6 PD. Enrichment of Tcm in the infused cells has improved the clinical outcome (CR/SD *vs*. PD, p=0.0464) (10). The used phenotype is CD45RO+CD62L+CCR7+.

38 patients have received CD19 CAR-T cell therapy for chronic lymphocytic leukemia. To produce CAR-T cells, PBMCs have been activated with anti-CD3 and anti-CD28 monoclonal-antibody-coated polystyrene beads, transduced with lentiviral vector, and expanded for 9-11 days. 8 patients have achieved CR, and 3 PR_TD_ (partial response with transformed disease), 5 PR, 25 NR. Objective response (CR/PR_TD_ *vs*. PR/NR) has associated with the frequency of CD8+CD45RO-CD27+ T cells (p=0.0009). Theoretically this phenotype involves Tn and Tscm. However, after substantial ex-vivo transduction and expansion, the likelihood of infusing gene-modified Tn cells is negligible. So it is mainly Tscm. Frequency of CD8+ Tscm from CR/PR_TD_ patients at the time of leukapheresis has been significantly higher than that in PR/NR patients (p=0.0055) (5). And likewise, CR higher than NR (p=0.0008), CR higher than PR (p=0.0121). This phenotype is CD8+CD45RO-CCR7+CD45RA+CD62L+CD27+CD28+ IL7Rα+CD95+.

25 patients have received 1-5×10^7^ or 1-5×10^8^ anti-B cell maturation antigen (BCMA) CAR-T cells for relapsed/refractory multiple myeloma. To produce CAR-T cells, autologous T cells have been stimulated with paramagnetic polystyrene beads coated with anti-CD3 and anti-CD28 monoclonal antibodies, transduced with a lentiviral vector, and expanded for 10-12 days with rhIL-2. 1 patient has achieved stringent CR, and 1 CR, 5 very good PR, 5 PR, 5 minimal response, 6 SD, 2 PD. A higher proportion of CD8+CD45RO-CD27+ T cell phenotype has been significantly associated with response (≥PR *vs*. <PR, p=0.0121) (8). This phenotype involves mainly Tscm.

16 patients with metastatic pancreatic carcinoma have received 1-3 cycles of CAR-T cell infusion. The dose range is 1.31-8.9×10^6^/kg. To produce CAR-T cells, autologous PBMCs have been stimulated with OKT3 and cultured in GT-T551 medium with 0.5% autologous serum and 500U/ml rhIL-2, transduced by anti-EGFR lentivirus after 2 days, and further expanded for 10 days in culture bags. 2 has lost follow-up. 4 patients have achieved PR, and 8 SD, 2 PD. Tcm numbers in infused CAR T cells have been significantly different between patients who achieved PR/SD and those who had PD (p=0.0468) (11). The used phenotype is CD45RO+CD62L+CCR7+.

38 patients with metastatic melanoma have received a single infusion of autologous unselected TILs. The dose range is 1.1×10^8^-1×10^11^ cells. TILs have been initially cultured from enzymatic tumor digests and tumor fragments produced by sharp dissection. Infusion products have been generated by rapid expansion with irradiated PBMC, anti-CD3 antibody, and IL-2 in a 1 to 1 mixture of CM and AIM-V medium supplemented with 5% human AB serum, and harvested on day 14. 17 patients have achieved CR, and 21 NR. The percentages of stem-like T cells in CR infusion products have been significantly higher than those in NR ones (p=0.0096) (100). The used phenotype is CD39-CD69-.

Among 24 patients with large B cell lymphoma (LBCL), there were 16 diffuse LBCL, 6 transformed follicular lymphoma, and 2 primary mediastinal B cell lymphoma. They have been treated with anti-CD19 CAR T cell. The dose is 2× 10^6^ CAR positive cells/kg. At 3-month follow-up, 9 have achieved CR (38%), 1 PR (4%), 13 PD (50%), and 1 not evaluable (NE) due to death from sepsis. Enrichment of CD8 memory T cells, including Tscm and Tcm, in the infused cells has improved the clinical outcome (101). The used phenotype is CD8+CCR7+CD27+.

However, there is a trial where analyzed phenotypes have shown no significant relevance to therapeutic outcomes. 36 patients with metastatic melanoma have received 1.5-107×10^9^ TCR-T cells infused intravenously followed by 720,000 U/kg IL-2 every 8 hours to tolerance. To produce TCR-T cells, autologous peripheral blood lymphocytes have been stimulated for 2 days at 10^6^/mL with 50 ng/mL OKT3 in complete AIMV media supplemented with 5% human serum and 300 IU IL-2, and transduced. On day 9-12, cells have been expanded for an additional 9-14 days in 6000 IU IL-2 with 50 ng/mL OKT-3 and irradiated allogeneic PBL feeder cells. 1 patient has achieved CR, and 8 PR, 27 NR. No substantial differences have been found in the cell phenotype between responding and nonresponding patients (6). The used phenotypic molecules include CD27, CD28, CD45RA and CD45RO. It may be because CD28+, CD45RA+ or CD45RO+ phenotypes contain the interference from more differentiated T cells such as Tem, Teff and Temra (58). This just from the opposite angle indicates the less differentiated phenotypes in better T-cell therapeutic outcomes. For CD27+ phenotype, it may be because the infused cell numbers have not been compared, but just the percentages have. Also, CD8 has not been included, different from the above-described trial using CD8+CD27+ phenotype (9). So, it may have been interfered by other subtypes such as Treg.

## Future Directions

### The Most Clinically Relevant T-Cell Phenotypic Molecules

**Figure 2:**
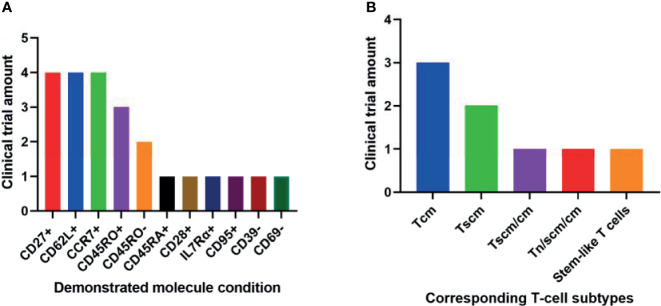
Phenotypic molecules **(A)** and T-cell subtype combinations **(B)** that have been demonstrated significantly associated with anti-cancer therapeutic outcomes in different amounts of clinical trials.

Among the 11 conditions, CD27+, CCR7+, and CD62L+ have been demonstrated by the most clinical trials in better outcomes of T-cell therapy (**Figure 2A**). These 3 molecules involve all the least differentiated subtypes including Tn, Tscm and Tcm. Hence, they have been most frequently used in clinical research.

It seems contradictory that both CD45RO+ and CD45RO- have been demonstrated in respectively 3 and 2 trials associated with better clinical outcomes (**Figure 2A**). They correspond to Tscm and Tcm respectively. Both subtypes have played important roles in T-cell therapy. So it is still reasonable these two opposite conditions have been implied in clinical trials.

However, individual phenotypic molecules hardly distinguish T cell subsets and differentiation status. There should be firstly dynamic integration between these phenotypic molecules before applying them to assess T cell differentiation. Also, with inherent substantial differences with TILs and gene-modified T cells, it might not be easy to compare phenotypical properties among them. Besides, clinical trial amount is limited as well, so the clinically relevant analysis here is currently just an indication.

### The Most Clinically Relevant T Cell Subtypes

The 2 least differentiated subtypes, Tscm and Tcm, have been demonstrated significantly associated with better clinical outcomes in various anti-cancer trials (**Figure 2B**). They are all important in T-cell therapy. Their relative importance may be due to different specific circumstances.

However, taking into consideration the heterogenous nature of T cells from different trials and the limited number of the trials, the most clinically relevant T cell subtypes addressed here are just implications. Future development of a universal definition of T cell subsets may strongly facilitate comparisons and meta-analysis for more clinically relevant T cell subtype.

### Differentiation Phenotypes to Evaluate Strategies of Enhancing T-Cell Stemness

Phenotypic analysis is effective and efficient to reveal T-cell products’ differentiation status, which is very important for therapeutic outcomes. Various strategies, including metabolites, epigenetic inhibitors, nutrient small molecules, and additional genetic engineering, have been shown to enhance T-cell stemness and anti-cancer therapy (3, 4, 58). But investigators still need to be very cautious in choosing phenotypic molecules. One cannot well develop strategies enhancing T-cell stemness without an efficient and stable differentiation phenotype. To the best of our knowledge, CD27+ CD62L+ and CCR7+ are currently recommended to be included in the differentiation phenotype.

Single-cell high-throughput technologies, including single-cell transcriptomics, have discovered new subtypes such as slowly dividing T central memory precursors (CMPs) and rapidly dividing non-CMPs (102). The discovery of more clinically relevant T cell subtype or phenotype may strongly improve T-cell production and anti-cancer therapy.

### Phenotypic Characteristics for Prognosis of T Cell Therapy

An efficient differentiation phenotype is important not only for research & development, but also for prognosis of T cell therapy. Before T cell infusion, the differentiation phenotype of the cell product can be analyzed to predict the potential clinical outcome. It may also be used to assess whether a cancer patient is suitable for the specific kind of T-cell therapy or T-cell production. Future studies may aim to discover highly clinically relevant differentiation phenotypes for specific T-cell production methods or specific subtypes of cancer patients, with the advantages of precision medicine.

In summary, differentiation status is crucial for T cell exhaustion, in-vivo lifespan, antitumor immunity, and even antitumor pharmacological interventions. Strategies including cytokines, gut microbiota, Akt signaling, epigenetics and metabolites have been developed to produce less differentiated T cells. Clinical trials have shown that infusion of T cells with less differentiated phenotypes achieves better clinical outcomes. Cancer-specific Tscm or Tcm cells, which express CD27, CCR7, and CD62L molecules, may be the most clinically relevant subtypes for successful T cell-based immunotherapy.

## Author Contributions

HR obtained the funding, did the literature review and wrote the article. KC did the literature review and drafted some parts. MW obtained the funding, revised the article and supervised this work. All authors contributed to the article and approved the submitted version.

## Funding

This work was supported by Shenzhen Basic Research Program (JCYJ20180507182902330, JCYJ20190809115811354), Program for Undertaking Major National Science and Technology Projects by Shenzhen (CJGJZD20200617102403009), Shenzhen Peacock Plan (KQTD20130416114522736), Special Funds for Dapeng New District Industry Development (KJYF202001-13, KJYF202001-12, KY20180214, PT201901-12), National Natural Science Foundation of China (32000975), and Guangdong Basic and Applied Basic Research Foundation (2020B1515120018, 2019A1515010629).

## Conflict of Interest

The authors declare that the research was conducted in the absence of any commercial or financial relationships that could be construed as a potential conflict of interest.

## Publisher’s Note

All claims expressed in this article are solely those of the authors and do not necessarily represent those of their affiliated organizations, or those of the publisher, the editors and the reviewers. Any product that may be evaluated in this article, or claim that may be made by its manufacturer, is not guaranteed or endorsed by the publisher.

## References

[1] Johnson LA June CH. Driving Gene-Engineered T Cell Immunotherapy of Cancer. Cell Res (2017) 27(1):38–58. doi: 10.1038/cr.2016.154 28025979 PMC5223234

[2] Wang RF Wang HY. Immune Targets and Neoantigens for Cancer Immunotherapy and Precision Medicine. Cell Res (2017) 27(1):11–37. doi: 10.1038/cr.2016.155 28025978 PMC5223235

[3] Liu Q Sun Z Chen L. Memory T Cells: Strategies for Optimizing Tumor Immunotherapy. Protein Cell (2020) 11(8):549–64. doi: 10.1007/s13238-020-00707-9 PMC7381543 32221812

[4] Busch DH Fräßle SP Sommermeyer D Buchholz VR Riddell SR. Role of Memory T Cell Subsets for Adoptive Immunotherapy. Semin Immunol (2016) 28(1):28–34. doi: 10.1016/j.smim.2016.02.001 26976826 PMC5027130

[5] Fraietta JA Lacey SF Orlando EJ Pruteanu-Malinici I Gohil M Lundh S. Determinants of Response and Resistance to CD19 Chimeric Antigen Receptor (CAR) T Cell Therapy of Chronic Lymphocytic Leukemia. Nat Med (2018) 24(5):563–71. doi: 10.1038/s41591-018-0010-1 PMC6117613 29713085

[6] Johnson LA Morgan RA Dudley ME Cassard L Yang JC Hughes MS. Gene Therapy With Human and Mouse T-Cell Receptors Mediates Cancer Regression and Targets Normal Tissues Expressing Cognate Antigen. Blood (2009) 114(3):535–46. doi: 10.1182/blood-2009-03-211714 PMC2929689 19451549

[7] Louis CU Savoldo B Dotti G Pule M Yvon E Myers GD. Antitumor Activity and Long-Term Fate of Chimeric Antigen Receptor-Positive T Cells In Patients With Neuroblastoma. Blood (2011) 118(23):6050–6. doi: 10.1182/blood-2011-05-354449 PMC3234664 21984804

[8] Cohen AD Garfall AL Stadtmauer EA Melenhorst JJ Lacey SF Lancaster E. B Cell Maturation Antigen-Specific CAR T Cells Are Clinically Active in Multiple Myeloma. J Clin Invest (2019) 129(6):2210–21. doi: 10.1172/JCI126397 PMC6546468 30896447

[9] Rosenberg SA Yang JC Sherry RM Kammula US Hughes MS Phan GQ. Durable Complete Responses in Heavily Pretreated Patients With Metastatic Melanoma Using T-Cell Transfer Immunotherapy. Clin Cancer Res (2011) 17(13):4550–7. doi: 10.1158/1078-0432.CCR-11-0116 PMC3131487 21498393

[10] Guo Y Feng K Liu Y Wu Z Dai H Yang Q. Phase I Study of Chimeric Antigen Receptor-Modified T Cells in Patients With EGFR-Positive Advanced Biliary Tract Cancers. Clin Cancer Res (2018) 24(6):1277–86. doi: 10.1158/1078-0432.CCR-17-0432 29138340

[11] Liu Y Guo Y Wu Z Feng K Tong C Wang Y. Anti-EGFR Chimeric Antigen Receptor-Modified T Cells in Metastatic Pancreatic Carcinoma: A Phase I Clinical Trial. Cytotherapy (2020) 22(10):573–80. doi: 10.1016/j.jcyt.2020.04.088 32527643

[12] Choi H Song H Jung YW. The Roles of CCR7 for the Homing of Memory CD8+ T Cells Into Their Survival Niches. Immune Netw (2020) 20(3):e20. doi: 10.4110/in.2020.20.e20 32655968 PMC7327150

[13] Moschovakis GL Förster R. Multifaceted Activities of CCR7 Regulate T-Cell Homeostasis in Health and Disease. Eur J Immunol (2012) 42(8):1949–55. doi: 10.1002/eji.201242614 22700449

[14] Grant EJ Nüssing S Sant S Clemens EB Kedzierska K. The Role of CD27 in Anti-Viral T-Cell Immunity. Curr Opin Virol (2017) 22:77–88. doi: 10.1016/j.coviro.2016.12.001 28086150

[15] Borst J Hendriks J Xiao Y. CD27 and CD70 in T Cell and B Cell Activation. Curr Opin Immunol (2005) 17(3):275–81. doi: 10.1016/j.coi.2005.04.004 15886117

[16] van de Ven K Borst J. Targeting the T-Cell Co-Stimulatory CD27/CD70 Pathway in Cancer Immunotherapy: Rationale and Potential. Immunotherapy (2015) 7(6):655–67. doi: 10.2217/imt.15.32 26098609

[17] Alegre ML Frauwirth KA Thompson CB. T-Cell Regulation by CD28 and CTLA-4. Nat Rev Immunol (2001) 1(3):220–8. doi: 10.1038/35105024 11905831

[18] Porciello N Tuosto L. CD28 Costimulatory Signals in T Lymphocyte Activation: Emerging Functions Beyond a Qualitative and Quantitative Support to TCR Signalling. Cytokine Growth Factor Rev (2016) 28:11–9. doi: 10.1016/j.cytogfr.2016.02.004 26970725

[19] Linsley PS Ledbetter JA. The Role of the CD28 Receptor During T Cell Responses to Antigen. Annu Rev Immunol (1993) 11:191–212. doi: 10.1146/annurev.iy.11.040193.001203 8386518

[20] Vallejo AN. CD28 Extinction in Human T Cells: Altered Functions and the Program of T-Cell Senescence. Immunol Rev (2005) 205:158–69. doi: 10.1111/j.0105-2896.2005.00256.x 15882352

[21] Huff WX Kwon JH Henriquez M Fetcko K Dey M. The Evolving Role of CD8(+)CD28(-) Immunosenescent T Cells in Cancer Immunology. Int J Mol Sci (2019) 20(11):2810. doi: 10.3390/ijms20112810 PMC6600236 31181772

[22] Mou D Espinosa J Lo DJ Kirk AD. CD28 Negative T Cells: Is Their Loss Our Gain? Am J Transplant (2014) 14(11):2460–6. doi: 10.1111/ajt.12937 PMC4886707 25323029

[23] Young A Mittal D Stagg J Smyth MJ. Targeting Cancer-Derived Adenosine: New Therapeutic Approaches. Cancer Discov (2014) 4(8):879–88. doi: 10.1158/2159-8290.CD-14-0341 25035124

[24] Gupta PK Godec J Wolski D Adland E Yates K Pauken KE. CD39 Expression Identifies Terminally Exhausted CD8+ T Cells. PLoS Pathog (2015) 11(10):e1005177. doi: 10.1371/journal.ppat.1005177 26485519 PMC4618999

[25] Canale FP Ramello MC Nunez N Araujo FC Bossio SN Gorosito SM. CD39 Expression Defines Cell Exhaustion in Tumor-Infiltrating CD8(+) T Cells. Cancer Res (2018) 78(1):115–28. doi: 10.1158/0008-5472.CAN-16-2684 29066514

[26] Sade-Feldman M Yizhak K Bjorgaard SL Ray JP de Boer CG Jenkins RW. Defining T Cell States Associated With Response to Checkpoint Immunotherapy in Melanoma. Cell (2018) 175(4):998–1013. doi: 10.1016/j.cell.2018.10.038 30388456 PMC6641984

[27] Allard B Pommey S Smyth MJ Stagg J. Targeting CD73 Enhances the Antitumor Activity of Anti-PD-1 and Anti-CTLA-4 Mabs. Clin Cancer Res (2013) 19(20):5626–35. doi: 10.1158/1078-0432.CCR-13-0545 23983257

[28] Thomas ML. The Leukocyte Common Antigen Family. Annu Rev Immunol (1989) 7:339–69. doi: 10.1146/annurev.iy.07.040189.002011 2523715

[29] Charbonneau H Tonks NK Walsh KA Fischer KA. The Leukocyte Common Antigen (CD45): A Putative Receptor-Linked Protein Tyrosine Phosphatase. Proc Natl Acad Sci USA (1988) 85(19):7182–6. doi: 10.1073/pnas.85.19.7182 PMC282148 2845400

[30] Barclay AN McCall MN. CD45; From Alloantigen to Mapping of Restricted Epitopes Using Recombinant Soluble CD45 Isoforms. Biochem Soc Trans (1992) 20(1):161–4. doi: 10.1042/bst0200161 1378795

[31] Hermiston ML Xu Z Weiss A. CD45: A Critical Regulator of Signaling Thresholds in Immune Cells. Annu Rev Immunol (2003) 21:107–37. doi: 10.1146/annurev.immunol.21.120601.140946 12414720

[32] Akbar AN Terry L Timms A Beverley PC Janossy G. Loss of CD45R and Gain of UCHL1 Reactivity Is a Feature of Primed T Cells. J Immunol (1988) 140(7):2171–8. 2965180

[33] Wills MR Carmichael AJ Weekes MP Mynard K Okecha G Hicks R. Human Virus-Specific CD8+ CTL Clones Revert From CD45ROhigh to CD45RAhigh In Vivo: CD45RAhighCD8+ T Cells Comprise Both Naive and Memory Cells. J Immunol (1999) 162(12):7080–7. 10358151

[34] Henson SM Riddell NE Akbar AN. Properties of End-Stage Human T Cells Defined by CD45RA Re-Expression. Curr Opin Immunol (2012) 24(4):476–81. doi: 10.1016/j.coi.2012.04.001 22554789

[35] von Andrian UH Mempel TR. Homing and Cellular Traffic in Lymph Nodes. Nat Rev Immunol (2003) 3(11):867–78. doi: 10.1038/nri1222 14668803

[36] Reinhardt RL Khoruts A Merica R Zell T Jenkins MK. Visualizing the Generation of Memory CD4 T Cells in the Whole Body. Nature (2001) 410(6824):101–5. doi: 10.1038/35065111 11242050

[37] Masopust D Vezys V Marzo AL Lefrancois L. Preferential Localization of Effector Memory Cells in Nonlymphoid Tissue. Science (2001) 291(5512):2413–7. doi: 10.1126/science.1058867 11264538

[38] Yamashita I Nagata T Tada T Nakayama T. CD69 Cell Surface Expression Identifies Developing Thymocytes Which Audition for T Cell Antigen Receptor-Mediated Positive Selection. Int Immunol (1993) 5(9):1139–50. doi: 10.1093/intimm/5.9.1139 7902130

[39] Ziegler SF Ramsdell F Alderson MR. The Activation Antigen CD69. Stem Cells (1994) 12(5):456–65. doi: 10.1002/stem.5530120502 7804122

[40] Shinoda K Tokoyoda K Hanazawa A Hayashizaki K Zehentmeier S Hosokawa H. Type II Membrane Protein CD69 Regulates the Formation of Resting T-Helper Memory. Proc Natl Acad Sci (2012) 109(19):7409–14. doi: 10.1073/pnas.1118539109 PMC3358871 22474373

[41] Shiow LR Rosen DB Brdickova N Xu Y An J Lanier LL. CD69 Acts Downstream of Interferon-Alpha/Beta to Inhibit S1P1 and Lymphocyte Egress From Lymphoid Organs. Nature (2006) 440(7083):540–4. doi: 10.1038/nature04606 16525420

[42] Matloubian M Lo CG Cinamon G Lesneski MJ Xu Y Brinkmann V. Lymphocyte Egress From Thymus and Peripheral Lymphoid Organs Is Dependent on S1P Receptor 1. Nature (2004) 427(6972):355–60. doi: 10.1038/nature02284 14737169

[43] Nakayama T Kasprowicz DJ Yamashita M Schubert LA Gillard G Kimura M. The Generation of Mature, Single-Positive Thymocytes In Vivo Is Dysregulated by CD69 Blockade or Overexpression. J Immunol (2002) 168(1):87–94. doi: 10.4049/jimmunol.168.1.87 11751950

[44] Vuckovic S Bryant CE Lau K Yang S Favaloro J McGuire HM. Inverse Relationship Between Oligoclonal Expanded CD69- TTE and CD69+ TTE Cells in Bone Marrow of Multiple Myeloma Patients. Blood Adv (2020) 4(19):4593–604. doi: 10.1182/bloodadvances.2020002237 PMC7556150 32986791

[45] Dulic S Toldi G Sava F Kovacs L Molnar T Milassin A. Specific T-Cell Subsets Can Predict the Efficacy of Anti-TNF Treatment in Inflammatory Bowel Diseases. Arch Immunol Ther Exp (Warsz) (2020) 68(2):12. doi: 10.1007/s00005-020-00575-5 32248339 PMC7128008

[46] Bouillet P O'Reilly LA. CD95, BIM and T Cell Homeostasis. Nat Rev Immunol (2009) 9(7):514–9. doi: 10.1038/nri2570 19543226

[47] Krueger A Fas SC Baumann S Krammer PH. The Role of CD95 in the Regulation of Peripheral T-Cell Apoptosis. Immunol Rev (2003) 193:58–69. doi: 10.1034/j.1600-065X.2003.00047.x 12752671

[48] Leonardi AJ Proenca RB. Akt-Fas to Quell Aberrant T Cell Differentiation and Apoptosis in Covid-19. Front Immunol (2020) 11:600405. doi: 10.3389/fimmu.2020.600405 33408715 PMC7779612

[49] von Freeden-Jeffry U Vieira P Lucian LA McNeil T Burdach SE Murray R. Lymphopenia in Interleukin (IL)-7 Gene-Deleted Mice Identifies IL-7 as a Nonredundant Cytokine. J Exp Med (1995) 181(4):1519–26. doi: 10.1084/jem.181.4.1519 PMC2191954 7699333

[50] Carrette F Surh CD. IL-7 Signaling and CD127 Receptor Regulation in the Control of T Cell Homeostasis. Semin Immunol (2012) 24(3):209–17. doi: 10.1016/j.smim.2012.04.010 PMC3367861 22551764

[51] Sprent J Surh CD. Normal T Cell Homeostasis: The Conversion of Naive Cells Into Memory-Phenotype Cells. Nat Immunol (2011) 12(6):478–84. doi: 10.1038/ni.2018 PMC3434123 21739670

[52] Parretta E Cassese G Santoni A Guardiola J Vecchio A Di Rosa F. Kinetics of In Vivo Proliferation and Death of Memory and Naive CD8 T Cells: Parameter Estimation Based on 5-Bromo-2'-Deoxyuridine Incorporation in Spleen, Lymph Nodes, and Bone Marrow. J Immunol (2008) 180(11):7230–9. doi: 10.4049/jimmunol.180.11.7230 18490722

[53] Philip M Fairchild L Sun L Horste EL Camara S Shakiba M. Chromatin States Define Tumour-Specific T Cell Dysfunction and Reprogramming. Nature (2017) 545(7655):452–6. doi: 10.1038/nature22367 PMC5693219 28514453

[54] Wu T Ji Y Moseman EA Xu HC Manglani M Kirby M. The TCF1-Bcl6 Axis Counteracts Type I Interferon to Repress Exhaustion and Maintain T Cell Stemness. Sci Immunol (2016) 1(6):eaai8593. doi: 10.1126/sciimmunol.aai8593 28018990 PMC5179228

[55] Smith-Garvin JE Koretzky GA Jordan MS. T Cell Activation. Annu Rev Immunol (2009) 27:591–619. doi: 10.1146/annurev.immunol.021908.132706 19132916 PMC2740335

[56] Gattinoni L Klebanoff CA Restifo NP. Paths to Stemness: Building the Ultimate Antitumour T Cell. Nat Rev Cancer (2012) 12(10):671–84. doi: 10.1038/nrc3322 PMC6352980 22996603

[57] Crompton JG Narayanan M Cuddapah S Roychoudhuri R Ji Y Yang W. Lineage Relationship of CD8(+) T Cell Subsets Is Revealed by Progressive Changes in the Epigenetic Landscape. Cell Mol Immunol (2016) 13(4):502–13. doi: 10.1038/cmi.2015.32 PMC4947817 25914936

[58] Kishton RJ Sukumar M Restifo NP. Metabolic Regulation of T Cell Longevity and Function in Tumor Immunotherapy. Cell Metab (2017) 26(1):94–109. doi: 10.1016/j.cmet.2017.06.016 28683298 PMC5543711

[59] Weng NP Levine BL June CH Hodes RJ. Human Naive and Memory T Lymphocytes Differ in Telomeric Length and Replicative Potential. Proc Natl Acad Sci USA (1995) 92(24):11091–4. doi: 10.1073/pnas.92.24.11091 PMC40577 7479943

[60] Gattinoni L Zhong XS Palmer DC Ji Y Hinrichs CS Yu Z. Wnt Signaling Arrests Effector T Cell Differentiation and Generates CD8+ Memory Stem Cells. Nat Med (2009) 15(7):808–13. doi: 10.1038/nm.1982 PMC2707501 19525962

[61] Gattinoni L Lugli E Ji Y Pos Z Paulos CM Quigley MF. A Human Memory T Cell Subset With Stem Cell-Like Properties. Nat Med (2011) 17(10):1290–7. doi: 10.1038/nm.2446 PMC3192229 21926977

[62] Robbins PF Dudley ME Wunderlich J El-Gamil M Li YF Zhou J. Cutting Edge: Persistence of Transferred Lymphocyte Clonotypes Correlates With Cancer Regression in Patients Receiving Cell Transfer Therapy. J Immunol (2004) 173(12):7125–30. doi: 10.4049/jimmunol.173.12.7125 PMC2175171 15585832

[63] Gattinoni L Klebanoff CA Palmer DC Wrzesinski DC Kerstann DC Yu Z. Acquisition of Full Effector Function In Vitro Paradoxically Impairs the In Vivo Antitumor Efficacy of Adoptively Transferred CD8+ T Cells. J Clin Invest (2005) 115(6):1616–26. doi: 10.1172/JCI24480 PMC1137001 15931392

[64] Klebanoff CA Gattinoni L Restifo NP. CD8+ T-Cell Memory in Tumor Immunology and Immunotherapy. Immunol Rev (2006) 211:214–24. doi: 10.1111/j.0105-2896.2006.00391.x PMC1501075 16824130

[65] Berger C Jensen MC Lansdorp PM Gough M Elliott C Riddell SR. Adoptive Transfer of Effector CD8+ T Cells Derived From Central Memory Cells Establishes Persistent T Cell Memory in Primates. J Clin Invest (2008) 118(1):294–305. doi: 10.1172/JCI32103 18060041 PMC2104476

[66] Huang J Khong HT Dudley ME El-Gamil M Li YF Rosenberg SA. Survival, Persistence, and Progressive Differentiation of Adoptively Transferred Tumor-Reactive T Cells Associated With Tumor Regression. J Immunother (2005) 28(3):258–67. doi: 10.1097/01.cji.0000158855.92792.7a PMC2174599 15838383

[67] Yang J Brook MO Carvalho-Gaspar M Zhang J Ramon HE Sayegh MH. Allograft Rejection Mediated by Memory T Cells Is Resistant to Regulation. Proc Natl Acad Sci USA (2007) 104(50):19954–9. doi: 10.1073/pnas.0704397104 PMC2148404 18042727

[68] Tyrakis PA Palazon A Macias D Lee KL Phan AT Velica P. S-2-Hydroxyglutarate Regulates CD8(+) T-Lymphocyte Fate. Nature (2016) 540(7632):236–41. doi: 10.1038/nature20165 PMC5149074 27798602

[69] Carrio R Bathe OF Malek TR. Initial Antigen Encounter Programs CD8+ T Cells Competent to Develop Into Memory Cells That Are Activated in an Antigen-Free, IL-7- and IL-15-Rich Environment. J Immunol (2004) 172(12):7315–23. doi: 10.4049/jimmunol.172.12.7315 15187107

[70] Adachi K Kano Y Nagai T Okuyama N Sakoda Y Tamada K. IL-7 and CCL19 Expression in CAR-T Cells Improves Immune Cell Infiltration and CAR-T Cell Survival in the Tumor. Nat Biotechnol (2018) 36(4):346–51. doi: 10.1038/nbt.4086 29505028

[71] Klarquist J Chitrakar A Pennock ND Kilgore AM Blain T Zheng C. Clonal Expansion of Vaccine-Elicited T Cells Is Independent of Aerobic Glycolysis. Sci Immunol (2018) 3(27):eaas9822. doi: 10.1126/sciimmunol.aas9822 30194241 PMC6251947

[72] Liu L Bi E Ma X Xiong W Qian J Ye L. Enhanced CAR-T Activity Against Established Tumors by Polarizing Human T Cells to Secrete Interleukin-9. Nat Commun (2020) 11(1):5902. doi: 10.1038/s41467-020-19672-2 33214555 PMC7677397

[73] Huang LR Chen FL Chen YT Lin YM Kung JT. Potent Induction of Long-Term CD8+ T Cell Memory by Short-Term IL-4 Exposure During T Cell Receptor Stimulation. Proc Natl Acad Sci USA (2000) 97(7):3406–11. doi: 10.1073/pnas.97.7.3406 PMC16252 10725381

[74] Ptáčková P Musil J Štach M Lesný P Němečková ` Král V. A New Approach to CAR T-Cell Gene Engineering and Cultivation Using Piggybac Transposon in the Presence of IL-4, IL-7 and IL-21. Cytotherapy (2018) 20(4):507–20. doi: 10.1016/j.jcyt.2017.10.001 29475789

[75] Merino D San SD Medina JM Rodrigo E Asensio E Irure J. Different In Vitro Proliferation and Cytokine-Production Inhibition of Memory T-Cell Subsets After Calcineurin and Mammalian Target of Rapamycin Inhibitors Treatment. Immunology (2016) 148(2):206–15. doi: 10.1111/imm.12603 PMC4863569 26931075

[76] Araki K Turner AP Shaffer VO Gangappa S Keller SA Bachmann MF. mTOR Regulates Memory CD8 T-Cell Differentiation. Nature (2009) 460(7251):108–12. doi: 10.1038/nature08155 PMC2710807 19543266

[77] Crompton JG Sukumar M Roychoudhuri R Clever D Gros A Eil RL. Akt Inhibition Enhances Expansion of Potent Tumor-Specific Lymphocytes With Memory Cell Characteristics. Cancer Res (2015) 75(2):296–305. doi: 10.1158/0008-5472.CAN-14-2277 25432172 PMC4384335

[78] Klebanoff CA Scott CD Leonardi AJ Yamamoto TN Cruz AC Ouyang C. Memory T Cell-Driven Differentiation of Naive Cells Impairs Adoptive Immunotherapy. J Clin Invest (2016) 126(1):318–34. doi: 10.1172/JCI81217 PMC4701537 26657860

[79] Eil R Vodnala SK Clever D Klebanoff CA Sukumar M Pan JH. Ionic Immune Suppression Within the Tumour Microenvironment Limits T Cell Effector Function. Nature (2016) 537(7621):539–43. doi: 10.1038/nature19364 PMC5204372 27626381

[80] Sabatino M Hu J Sommariva M Gautam S Fellowes V Hocker JD. Generation of Clinical-Grade CD19-Specific CAR-Modified CD8+ Memory Stem Cells for the Treatment of Human B-Cell Malignancies. Blood (2016) 128(4):519–28. doi: 10.1182/blood-2015-11-683847 PMC4965906 27226436

[81] Kondo T Morita R Okuzono Y Nakatsukasa H Sekiya T Chikuma S. Notch-Mediated Conversion of Activated T Cells Into Stem Cell Memory-Like T Cells for Adoptive Immunotherapy. Nat Commun (2017) 8:15338. doi: 10.1038/ncomms15338 28530241 PMC5458121

[82] Ghoneim HE Fan Y Moustaki A Abdelsamed HA Dash P Dogra P. De Novo Epigenetic Programs Inhibit PD-1 Blockade-Mediated T Cell Rejuvenation. Cell (2017) 170(1):142–57. doi: 10.1016/j.cell.2017.06.007 PMC5568784 28648661

[83] Takahashi K Yamanaka S. Induction of Pluripotent Stem Cells From Mouse Embryonic and Adult Fibroblast Cultures by Defined Factors. Cell (2006) 126(4):663–76. doi: 10.1016/j.cell.2006.07.024 16904174

[84] Chronis C Fiziev P Papp B Butz S Bonora G Sabri S. Cooperative Binding of Transcription Factors Orchestrates Reprogramming. Cell (2017) 168(3):442–59. doi: 10.1016/j.cell.2016.12.016 PMC5302508 28111071

[85] Zhang H Jiao W Sun L Fan J Chen M Wang H. Intrachromosomal Looping Is Required for Activation of Endogenous Pluripotency Genes During Reprogramming. Cell Stem Cell (2013) 13(1):30–5. doi: 10.1016/j.stem.2013.05.012 23747202

[86] Themeli M Riviere I Sadelain M. New Cell Sources for T Cell Engineering and Adoptive Immunotherapy. Cell Stem Cell (2015) 16(4):357–66. doi: 10.1016/j.stem.2015.03.011 PMC5611846 25842976

[87] Wei Z Gao F Kim S Yang H Lyu J An W. Klf4 Organizes Long-Range Chromosomal Interactions With the Oct4 Locus in Reprogramming and Pluripotency. Cell Stem Cell (2013) 13(1):36–47. doi: 10.1016/j.stem.2013.05.010 23747203

[88] Crompton JG Clever D Vizcardo R Rao M Restifo NP. Reprogramming Antitumor Immunity. Trends Immunol (2014) 35(4):178–85. doi: 10.1016/j.it.2014.02.003 PMC4373650 24661777

[89] Henning AN Klebanoff CA Restifo NP. Silencing Stemness in T Cell Differentiation. Science (2018) 359(6372):163–4. doi: 10.1126/science.aar5541 PMC6312704 29326263

[90] Kagoya Y Nakatsugawa M Yamashita Y Ochi T Guo T Anczurowski M. BET Bromodomain Inhibition Enhances T Cell Persistence and Function in Adoptive Immunotherapy Models. J Clin Invest (2016) 126(9):3479–94. doi: 10.1172/JCI86437 PMC5004946 27548527

[91] Losman JA Kaelin WJ. What a Difference a Hydroxyl Makes: Mutant IDH, (R)-2-Hydroxyglutarate, and Cancer. Genes Dev (2013) 27(8):836–52. doi: 10.1101/gad.217406.113 PMC3650222 23630074

[92] Gautam S Fioravanti J Zhu W Le Gall JB Brohawn P Lacey NE. The Transcription Factor C-Myb Regulates CD8+ T Cell Stemness and Antitumor Immunity. Nat Immunol (2019) 20(3):337–49. doi: 10.1038/s41590-018-0311-z PMC6489499 30778251

[93] Ji Y Fioravanti J Zhu W Wang H Wu T Hu J. miR-155 Harnesses Phf19 to Potentiate Cancer Immunotherapy Through Epigenetic Reprogramming of CD8+ T Cell Fate. Nat Commun (2019) 10(1):2157. doi: 10.1038/s41467-019-09882-8 31089138 PMC6517388

[94] Sukumar M Liu J Ji Y Subramanian M Crompton JG Yu Z. Inhibiting Glycolytic Metabolism Enhances CD8+ T Cell Memory and Antitumor Function. J Clin Invest (2013) 123(10):4479–88. doi: 10.1172/JCI69589 PMC3784544 24091329

[95] Geiger R Rieckmann JC Wolf T Basso C Feng Y Fuhrer T. L-Arginine Modulates T Cell Metabolism and Enhances Survival and Anti-Tumor Activity. Cell (2016) 167(3):829–42. doi: 10.1016/j.cell.2016.09.031 PMC5075284 27745970

[96] Hermans D Gautam S García-Cañaveras JC Gromer D Mitra S Spolski R. Lactate Dehydrogenase Inhibition Synergizes With IL-21 to Promote CD8(+) T Cell Stemness and Antitumor Immunity. Proc Natl Acad Sci USA (2020) 117(11):6047–55. doi: 10.1073/pnas.1920413117 PMC7084161 32123114

[97] Buck MD O'Sullivan D Klein GR Curtis JD Chang CH Sanin DE. Mitochondrial Dynamics Controls T Cell Fate Through Metabolic Programming. Cell (2016) 166(1):63–76. doi: 10.1016/j.cell.2016.05.035 27293185 PMC4974356

[98] Hu X Majchrzak K Liu X Wyatt MM Spooner CJ Moisan J. In Vitro Priming of Adoptively Transferred T Cells With a RORgamma Agonist Confers Durable Memory and Stemness In Vivo. Cancer Res (2018) 78(14):3888–98. doi: 10.1158/0008-5472.CAN-17-3973 PMC6238208 29769201

[99] Borges DSH Beura LK Wang H Hanse EA Gore R Scott MC. The Purinergic Receptor P2RX7 Directs Metabolic Fitness of Long-Lived Memory CD8(+) T Cells. Nature (2018) 559(7713):264–8. doi: 10.1038/s41586-018-0282-0 PMC6054485 29973721

[100] Krishna S Lowery FJ Copeland AR Bahadiroglu E Mukherjee R Jia L. Stem-Like CD8 T Cells Mediate Response of Adoptive Cell Immunotherapy Against Human Cancer. Science (2020) 370(6522):1328–34. doi: 10.1126/science.abb9847 PMC8883579 33303615

[101] Deng Q Han G Puebla-Osorio N Ma MCJ Strati P Chasen B. Characteristics of Anti-CD19 CAR T Cell Infusion Products Associated With Efficacy and Toxicity in Patients With Large B Cell Lymphomas. Nat Med (2020) 26(12):1878–87. doi: 10.1038/s41591-020-1061-7 PMC8446909 33020644

[102] Kretschmer L Busch DH Buchholz VR. A Single-Cell Perspective on Memory T-Cell Differentiation. Cold Spring Harb Perspect Biol (2021) 13(9):a038067. doi: 10.1101/cshperspect.a038067 33903160 PMC8411955

